# A novel heterozygous variant in *FGF9* associated with previously unreported features of multiple synostosis syndrome 3

**DOI:** 10.1111/cge.13880

**Published:** 2021-01-13

**Authors:** Ann‐Charlotte Thuresson, Brittany Croft, Yasmin D. Hailer, Gunnar Liminga, Carl‐Göran Arvidsson, Vincent R. Harley, Eva‐Lena Stattin

**Affiliations:** ^1^ Department of Immunology, Genetics and Pathology, Science for Life Laboratory Uppsala Uppsala University Uppsala Sweden; ^2^ Hudson Institute of Medical Research Monash Medical Centre Melbourne Australia; ^3^ Department of Molecular and Translational Science Monash University Melbourne Australia; ^4^ Section of Orthopaedics, Department of Surgical Sciences Uppsala University Sweden; ^5^ Department of Women and Children's Health, Paediatric Neurology Uppsala University Uppsala Sweden; ^6^ Department of Paediatrics Västmanland's Hospital Västerås Sweden

**Keywords:** FGF9, fusion of interphalangeal joints, multiple synostosis syndrome, SYNS

## Abstract

Human multiple synostoses syndrome 3 is an autosomal dominant disorder caused by pathogenic variants in *FGF9*. Only two variants have been described in *FGF9* in humans so far, and one in mice. Here we report a novel missense variant c.566C > G, p.(Pro189Arg) in *FGF9*. Functional studies showed this variant impairs FGF9 homodimerization, but not FGFR3c binding. We also review the findings of cases reported previously and report on additional features not described previously.

## Graphical abstract

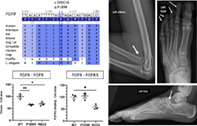

## 1 INTRODUCTION

Multiple synostosis syndrome (SYNS) is a heterogeneous group of rare autosomal dominant disorders, characterised by multiple joints fusions, facial features, and progressive conductive deafness. To date, four different types of SYNS associated with four different genes, have been described, SYNS1 (MIM#186500), SYNS2 (MIM#610017), SYNS3 (MIM#612961) and SYNS4 (MIM#617898), which are caused by heterozygous pathogenic variants in *NOG* (MIM#602991), *GDF5* (MIM#601146), *FGF9* (MIM#600921) and *GDF6* (MIM#601147), respectively. The different types of SYNS present with a similar phenotype, suggesting a common pathogenic mechanism and regulatory pathway.

To date, only two pathogenic missense variants associated with SYNS3 have been described in *FGF9* (NM_002010.2); c.296G > A; p.(Ser99Asn) (S99N) and c.184A > G; p.(Arg62Gly) (R62G).^1^, ^2^ In addition, a spontaneous missense variant, c.428A > C, p.(Asn143Thr) (N143T), was described in mouse (*Eks* mutant mouse) with a phenotype resembling SYNS3,^3^ and together with the S99N synostosis mutant mouse model, XY sex reversal.^4^

Here we report a boy with a novel likely pathogenic missense variant in *FGF9*, with previously unreported features of SYNS3 including bifid uvula, high‐narrow palate with crowded teeth, crossbite, large hands and feet with long fingers and toes.

## 2 CASE REPORT

A 16‐year old boy was referred to the department of clinical genetics due to suspected skeletal dysplasia. He was born at term, as the oldest child of three, to non‐consanguineous parents. There was no family history of skeletal malformations. Since early childhood, the patient showed a reduced range of motion in his elbows, thumbs and great toes. The parents reported minor deficits in fine motor skills. The patient complained of knee and ankle pain after physical activity.

Clinical examination showed pectus excavatum, a high‐narrow palate and bifid uvula. The patient wore dental braces due to crowded teeth and to crossbite, but also to widen the palate. He had large hands and feet, long fingers with bilateral fifth finger clinodactyly, decreased thumb flexion and bilateral slightly medially deviated hallux. He had an absence of skin creases over the distal interphalangeal joints of the toes, halluces and thumbs (Table 1). Mental development, growth, hearing and male genitalia (tanner 4) were normal.

**TABLE 1:** Clinical features presented in the present case and previously published cases with SYNS3

Cases (age)	Present case	Wu et al.^2^	Rodriguez‐Zabala et al.^1^	
	index (16 y)	12 individuals (age 5–68)	index (5 y)	Father (unknown)
*FGF9* (NM_002010.2)	c 566C > G; p.(Pro189Arg)	c.296G > A; p.(Ser99Asn)	c.184A > G; p.(Arg62Gly)	c.184A > G; p.(Arg62Gly)
Craniosynostosis	–		sagittal synostosis, dolichocephaly	suspected sagittal synostosis, dolichocephaly
Palate	high‐narrow palate and bifid uvula		−	cleft palate
Teeth	crowded	NA	NA	NA
Exophthalmos (Proptosis)	−		mild	+
Anomaly of the elbow joint	cubitus valgus, restricted elbow rotation	12/12	NA	
Broad thumbs	−		+	+ (radially deviated)
Synostosis of the digits	fusion of the interphalangeal joint of the thumb	10/12		congenital fixed contractures of the interphalangeal joints
Clinodactyly	5th finger			
Broad halluces	+ (medially deviated)			+ (medially deviated)
Synostosis of the toes	fusion of interphalangeal joints of toes	8/12		progressive movement limitation of the carpal, tarsal, interphalangeal of the toes
Synostosis of the vertebrae	L3‐L5	3/12		movement limitation of vertebral lumbar joints
X‐ray	osseous fusion of affected joints,			osseous fusion of affected joints
Other	pectus excavatum, radial bowing, compressed femoral condyle			

Abbreviation: NA, not applicable; +, present; −, absent.

**FIGURE 1:**
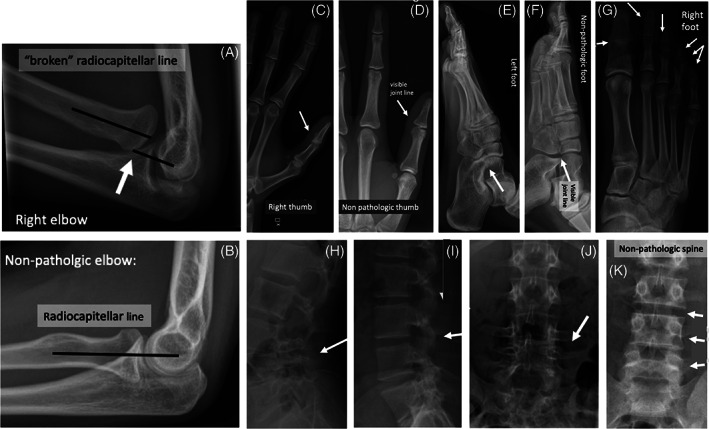
Radiographs showing the present patient with SYNS3 and a missense variant in FGF9. (A) Radial head dislocation of the elbow; (B) Normal elbow; (C) Synostosis of the interphalangeal joints of the thumb; (D) Normal thumb; (E) Suspected calcaneonavicular coalition of the feet; (F) Normal foot; (G) Synostosis of dig I and of the distal interphalangeal joints of dig II‐V of the feet; (H) Synostosis of the dorsal structures of the lumbar spine; (I, K) Well defined vertebrae and intervertebral joints of the lumbar spine and (J) Reduced height of the disc L3‐L4 and L4‐L5

Radiographs revealed a bilateral radial head dislocation of the elbows (Figure 1(A)) and partial bilateral fusion of the thumbs' interphalangeal joint (Figure 1(C)). A similar pattern was seen in the feet with a fusion of the interphalangeal joints of dig I and of the distal interphalangeal joints of dig II‐V (Figure 1(G)). Radiographs raised suspicion for a bilateral calcaneonavicular coalition (Figure 1(E)), and the lumbar spine showed a fusion of the dorsal portions of the vertebrae L3 to S1 with reduced height of the disc L3‐L4 and L4‐L5 (Figure 1(H), (J)).

Clinical findings indicated SYNS3, and DNA was sent for molecular genetic evaluation of the *FGF9* gene.

## 3 MATERIAL AND METHODS

### 3.1 Molecular genetic evaluation

Genomic DNA was extracted from peripheral blood leucocytes according to standard procedures. Sequencing and deletion/duplication analysis of *FGF9* was performed using a targeted NGS technology (Fulgent Genetics).

### 3.2 FGF9 dimerization and FGF9:FGFR3 binding

Human cDNA FGF9‐WT and FGF9‐R62G pcDNA3.1 HA and FLAG and FGFR3‐MYC tagged plasmids were kindly gifted from Dr Karen Heath.^1^ Modification to the wild type FGF9 cDNA sequences to introduce the P189R variant was introduced using the Agilent Site‐Directed mutagenesis kit with the primers P189R 5′‐CAGAAATTCACACATTTTTTACGTAGACCAGTGGACCCC‐3′ and 5’‐GGGGTCCACTGGTCTACGTAAAAAATGTGTGAATTTCTG‐3′. Expression plasmids were confirmed for the presence of FGF9 and FGFR3 cDNA as well as the engineered mutations using standard T7 primers and confirmed with Sanger sequencing at Monash Health and Translational Precinct Medical Genomics Facility.

Proximity ligation assay (PLA) was conducted using a modified protocol based off one kindly provided by Dr Karen Heath.^1^ Briefly, Human Embryonic Kidney cell line HEK293 cells were maintained in culture in DMEM, 10% FCS and 1% anti–anti at 37°C. Cells were dissociated using 0.025% trypsin. 45 000 HEK293 cells were seeded per Poly‐D‐lysine coated 8 well chamber slides overnight; cells were transfected with tagged FGF9 plasmids totalling 400 ng (eg, 200 ng FGF9‐HA and 200 ng FGF9‐FLAG) using lipofectamine 2000 (ThermoFisher) and incubated for a further 24 h. Cells were fixed in 4% PFA (15 min), blocked using Sigma Duolink PLA blocking buffer at room temperature (1 h) and stained with the required combination of rabbit anti‐HA (Abcam AB9110, 1:700), mouse anti‐c‐MYC (ThermoFisher 9E10.3, 1:1000) and mouse anti‐FLAG (Sigma‐Aldrich F1804, 1:200) primary antibodies at 4°C overnight. PLA signal was amplified using Sigma Duolink In Situ Orange Mouse/Rabbit kit (Sigma‐Aldrich) as per manufacturer's instructions. PLA signal was imaged on a confocal Olympus FV1200 and Nikon C1 (Monash Medical Imaging) under standard conditions (40x oil objective). The relative quantity of PLA signals was assessed using FIJI image software using the “analyse particles” function. Each biological replicate represents at least three image captures analysing the number of PLAs per cell area of at least 20 individual cells per condition, relative to wildtype. All statistical analysis was conducted in GraphPad Prism v8.1 (GraphPad Inc.) using appropriate tests as stated in figure captions.

Uppsala Ethical Review Board approved the study. Informed consent was obtained from the proband and his parents.

## 4 RESULTS

### 4.1 Molecular genetic evaluation

Sequencing identified a de novo novel heterozygous missense variant in *FGF9*, NM_002010.2:c.566C > G, p.(Pro189Arg). The variant could not be detected amongst >138 000 exome/genome sequences included in the gnomAD database (v2.1.1), is predicted as probably damaging by Polyphen2, disease casing by MutationTaster, deleterious by SIFT and has a CADD score of 28.7. The cytosine at nucleotide position 566 as well as the amino acid Pro189 are both highly conserved. Hence, the variant could thereby be classified as likely pathogenic.

### 4.2 FGF9 dimerization and FGF9:FGFR3c binding

**FIGURE 2:**
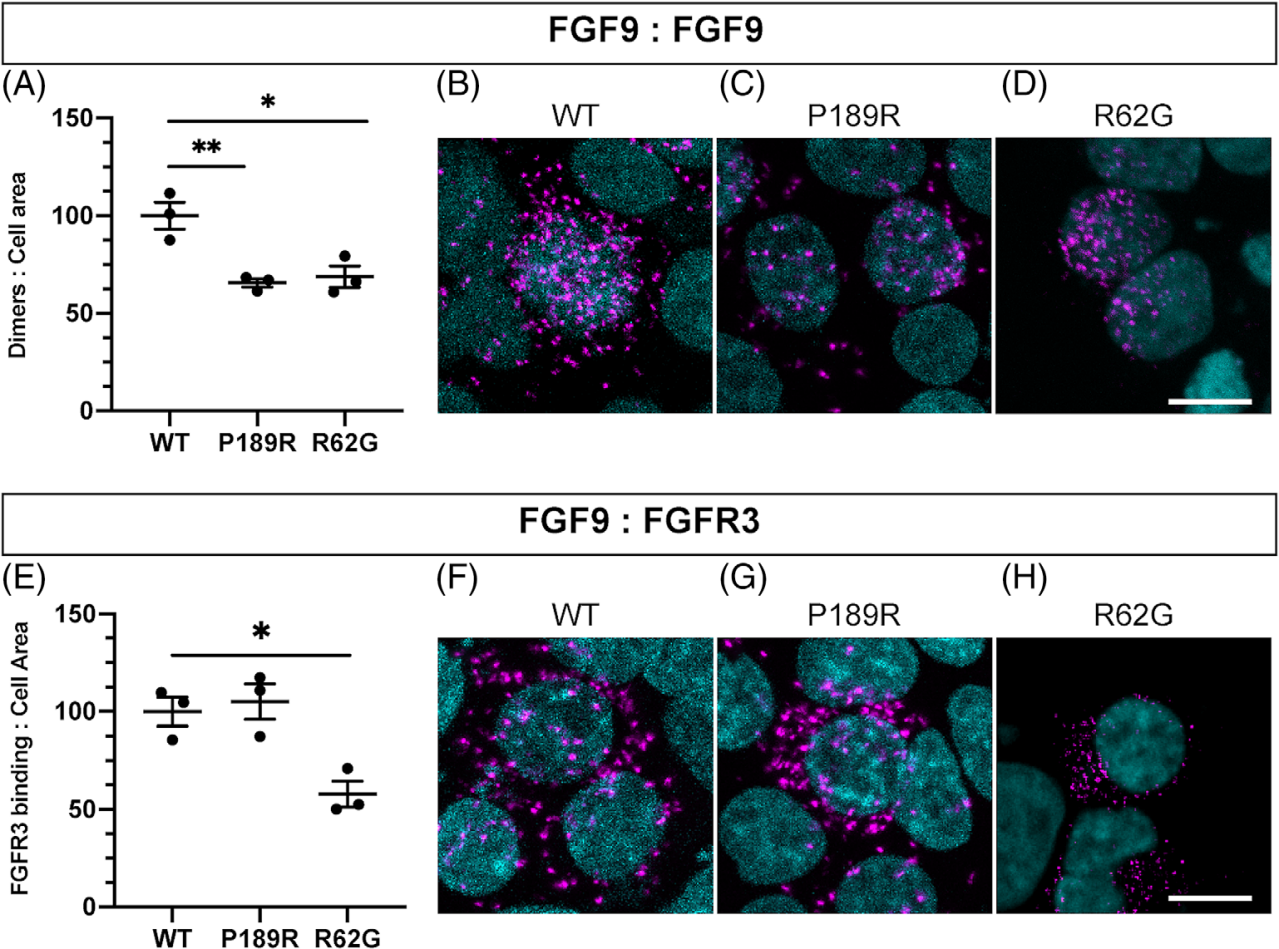
FGF9 homodimerization and quantification of FGF9:FGFR3 binding. (A) Quantification of FGF9 homodimerization events detected using PLA. All values were normalised to wildtype (WT) set at 100. (B‐C) Representative images of intercellular FGF9:FGF9 homodimerization of WT, P189R:P189R (P189R) and R62G:R62G (R62G)‐FGF9 variants. E, Quantification of FGF9 binding to FGFR3c events detected using PLA signals. All values were normalised to WT set at 100. (F‐H) Representative images of intercellular FGF9: FGFR3c interactions of WT, P189R and R62G‐FGF9 variants. Graphs represent the mean +/− SEM of n = 3 biological repeats (black dots),*p < 0.05 and **p < 0.01 using one way ANOVA with Dunnett's multiple comparison test. Representative images use a white scale bar 10um, DAPI‐cyan, PLA signals‐magenta dots [Colour figure can be viewed at wileyonlinelibrary.com]

Molecular modelling of the P189R variant predicted a P189 residue to be involved in FGF9 homodimerization and receptor binding.^5^ To determine whether the P189R substitution disrupted FGF9 homodimerization in a cellular context, in vitro PLAs were used. Mammalian expression plasmids harbouring epitope‐tagged wildtype or P189R mutant FGF9‐HA‐pcDNA3.1 and FGF9‐FLAG‐pcDNA3.1 were transiently transfected into HEK293‐T cells (Figure 2). The previously published synostosis causing *FGF9* variant R62G was used as a control,^1^ as this is the only variant known to both impede homodimerization and FGFR3 binding. FGF9 homodimerization events were detected for all variants and controls (Figure 2(B)–(D)). Relative to wildtype (WT) FGF9 HA‐FLAG, cells expressing P198R HA‐FLAG exhibited a reduced level of homodimerization (65.6% of WT) similar to the R62G control (68.9% of WT) (Figure 2(A)). Previous studies demonstrated that the R62G and S99N variants effects the affinity of FGF9 to FGFR3,^1^, ^2^ and that the N143T variant has an effect on FGFR3b but not FGFR3c.^3^ To test whether P189R alters FGF receptor binding, we again used PLAs. We focused our analysis on FGF9‐FGFR3c interactions using a MYC‐tagged FGFR3c‐PCMV mammalian expression plasmid (Figure 2(F)–(H)).^1^ Analysis of FGF9 P189R‐FGFR3c interaction revealed no loss of receptor binding (105.1% of WT) (Figure 2(E)). Whereas the R62G control had reduced receptor binding (57.8% of WT). Taken together, these results indicate the *FGF9* ^*P189R*^ variant disrupted FGF9 homodimerization and not receptor binding in the patient. Consequently, the variant detected in our patient could thereby be reclassified as pathogenic.

## 5 DISCUSSION

In this study we report a novel pathogenic variant in *FGF9*, c 566C > G, p.(Pro189Arg), in a patient with a phenotype highly specific for SYNS3. The detected variant impairs FGF9 homodimerization, but not FGFR3c binding.

Apart from the previously described clinical manifestations for SYNS3, like synostoses of elbow joints, hands, lumbar and feet,^1^, ^2^ the patient presented with crowded teeth, high‐narrow palate and bifid uvula, clinodactyly dig V and pectus excavatum (Table 1). Teeth abnormalities were not described in any of the previous cases, but abnormal teeth development was present in mice harbouring the S99N variant.^6^ The two cases described by Rodriguez‐Zabala et al. also presented with craniosynostosis and exophthalmos, not detected in our patient or the 12 cases described by Wu et al (Table 1). Hearing impairment has been described for SYNS1 and 4, but not for SYNS3 cases, including our case.

Functional studies of the previously reported variants in *FGF9* (R62G, S99N and N143T) showed that both the R62G variant and the Eks mutation N143T impair homodimerization, while the S99N variant does not.^1^, ^3^ Moreover, these studies also revealed that both the R62G and S99N variant ligands impede FGFR3 binding, while the Eks N143T ligand impairs binding to the FGFR3b isoform, but not to FGFR3c.^3^ Loss of dimerization also impairs heparin binding^5^ as seen in the e*ks* mutant.^3^ Mutations affecting the homodimerization likely inhibit FGF9 signalling by suppressing the FGFR3 binding and heparan sulphate‐dependent diffusion in the extracellular matrix.^5^

FGF9 is necessary for normal joint development, by regulating mesenchymal cell differentiation during embryogenesis.^3^, ^6^ Moreover, FGF9 regulates skeletal development and homeostasis by autoregulation.^6^ FGF9 signalling is dependent on the formation of a ternary complex between FGF9, fibroblast growth factor receptor (FGFR) and heparan sulphate proteoglycan. The formation of this complex is regulated by the ability of FGF9 to undergo reversible homodimerization.^7^ Reduced homodimerization of FGF9 will result eventually in higher concentrations of the active monomeric form of FGF9. In this way, the concentration of active FGF9 monomers is kept at a level sufficient for normal binding and signalling through FGFR3c, the isoform FGF9 most strongly binds. However, the concentration of FGF9 monomers is not sufficiently high to regulate the binding and activation of the FGFRb isoform.^7^ The FGF9 residues participating in the homodimerization also mediate the binding to other receptor classes such as FGFR3c.

The P189R variant detected in our patient, changes a neutral proline to a positively charged arginine. This modification might also cause a conformational change as these residues differ in size and proline is very rigid. The crystal structure and protein modelling of FGF9 indicate P189 residue interacts with the linker region of FGFRs, while the neighbouring amino acids, L188 and R190, interact with the protein domains of FGFR.^8^ This might suggest the P189 residue is not critical for receptor binding, consistent with our results. Alternatively, the P189R variant may impact binding to another isoform of FGFR.

Taken together, we present a third case of a novel variant in *FGF9* as the cause of SYNS3. Functional characterisation demonstrated this variant has an effect in vitro on homodimerization of FGF9 and will thereby most likely disturb autoregulation and joint development.

## CONFLICT OF INTEREST

The authors declare no conflict of interests.

### PEER REVIEW

The peer review history for this article is available at https://publons.com/publon/10.1111/cge.13880.

## Data Availability

Data analysed during this study are included in this published article. Raw data is available upon request.

## References

[1] Rodriguez‐Zabala M , Aza‐Carmona M , Rivera‐Pedroza CI , et al. FGF9 mutation causes craniosynostosis along with multiple synostoses. Hum Mutat. 2017;38:1471‐1476. 28730625 10.1002/humu.23292

[2] Wu XL , Gu MM , Huang L , et al. Multiple synostoses syndrome is due to a missense mutation in exon 2 of FGF9 gene. Am J Hum Genet. 2009;85:53‐63. 19589401 10.1016/j.ajhg.2009.06.007 PMC2706969

[3] Harada M , Murakami H , Okawa A , et al. FGF9 monomer‐dimer equilibrium regulates extracellular matrix affinity and tissue diffusion. Nat Genet. 2009;41:289‐298. 19219044 10.1038/ng.316 PMC2676118

[4] Bird AD , Croft BM , Harada M , et al. Ovotesticular disorders of sex development in FGF9 mouse models of human synostosis syndromes. Hum Mol Genet. 2020;29:2148‐2161. 32452519 10.1093/hmg/ddaa100

[5] Kalinina J , Byron SA , Makarenkova HP , et al. Homodimerization controls the fibroblast growth factor 9 subfamily's receptor binding and heparan sulfate‐dependent diffusion in the extracellular matrix. Mol Cell Biol. 2009;29:4663‐4678. 19564416 10.1128/MCB.01780-08 PMC2725704

[6] Tang L , Wu X , Zhang H , et al. A point mutation in Fgf9 impedes joint interzone formation leading to multiple synostoses syndrome. Hum Mol Genet. 2017;26:1280‐1293. 28169396 10.1093/hmg/ddx029

[7] Liu Y , Ma J , Beenken A , et al. Regulation of receptor binding specificity of FGF9 by an autoinhibitory homodimerization. Structure. 2017;25:1325‐1336 e1323. 28757146 10.1016/j.str.2017.06.016 PMC5587394

[8] Plotnikov AN , Eliseenkova AV , Ibrahimi OA , et al. Crystal structure of fibroblast growth factor 9 reveals regions implicated in dimerization and autoinhibition. J Biol Chem. 2001;276:4322‐4329. 11060292 10.1074/jbc.M006502200

